# A mouse ear skin model to study the dynamics of innate immune responses against the microsporidian *Encephalitozoon cuniculi*

**DOI:** 10.3389/fmicb.2023.1168970

**Published:** 2023-04-13

**Authors:** Eugénie Carriere, Aizat Iman Abdul Hamid, Inès Feki, Aurore Dubuffet, Frédéric Delbac, Pascale Gueirard

**Affiliations:** Laboratoire Microorganismes: Génome et Environnement, CNRS UMR 6023, Université Clermont Auvergne, Clermont-Ferrand, France

**Keywords:** microsporidia, *Encephalitozoon cuniculi*, inflammation, phagocytes, murine model, intravital imaging

## Abstract

Microsporidia are obligate intracellular parasites related to fungi that cause severe infections in immunocompromised individuals. *Encephalitozoon cuniculi* is a microsporidian species capable of infecting mammals, including human and rodents. In response to microsporidian infection, innate immune system serves as the first line of defense and allows a partial clearance of the parasite via the innate immune cells, namely macrophages, neutrophils, dendritic cells, and Natural Killer cells. According to the literature, microsporidia bypass this response *in vitro* by modulating the response of macrophages. In order to study host-parasites interactions *in vivo*, we developed a model using the mouse ear pinna in combination with an intravital imaging approach. Fluorescent *E. cuniculi* spores were inoculated into the skin tissue to follow for the first time in real time in an *in vivo* model the recruitment dynamics of EGFP + phagocytic cells in response to the parasite. The results show that parasites induce an important inflammatory recruitment of phagocytes, with alterations of their motility properties (speed, displacement length, straightness). This cellular response persists in the injection zone, with spores detected inside the phagocytes up to 72 h post-infection. Immunostainings performed on ear tissue cryosections evoke the presence of developing infectious foci from 5 days post-infection, in favor of parasite proliferation in this tissue. Overall, the newly set up mice ear pinna model will increase our understanding of the immunobiology of microsporidia and in particular, to know how they can bypass and hijack the host immune system of an immunocompetent or immunosuppressed host.

## 1. Introduction

Microsporidia are a phylum of fungus-like, obligate intracellular, spore-forming eukaryotic parasites that infect a very wide range of hosts, from invertebrates to vertebrates (Didier et al., 2004; Didier and Weiss, 2006). Of the 220 genera and 1,700 species that comprise the phylum Microsporidia, *Encephalitozoon cuniculi* is known to parasitize several mammals, including humans and rodents (Khan et al., 2001; Han et al., 2021). The general prevalence of microsporidiosis in humans is about 10%, with highly variable frequencies depending on the population considered (geographical origin, age groups, immune status, symptoms) or on the diagnostic method used (serology, molecular biology, microscopy) (Ruan et al., 2021). Infections typically occur through the gastrointestinal tract, but some Microsporidia, including *E. cuniculi*, can also disseminate to other organs (Didier and Weiss, 2011; Han et al., 2021). Clinical symptoms vary, depending on the immune status of the host (Didier, 2005). In immunocompromised individuals, infections result in severe consequences, with persistent diarrhea, malabsorption, and weight loss. In immunocompetent individuals, Microsporidia can survive asymptomatically, with a spontaneously resolving course of infection (Didier, 2005; Pan et al., 2018). However, cases of recurrent infections were also described, with parasite persistence within a tissue niche that has yet to be identified (Sak et al., 2011; Nourrisson et al., 2021). At the cellular level, *E. cuniculi* is known to infect non-professional phagocytes as epithelial cells, endothelial cells and fibroblasts, but also professional phagocytes as macrophages (Mo) (Dalboni et al., 2021). Parasites enter inside these cells after being phagocytosed. To actively invade host cells, microsporidian parasites have evolved a unique and highly specialized invasion apparatus, the protein-rich polar tube, which allows the transfer of infectious material, called sporoplasm, into the cytoplasm of the host cell (Cali and Takvorian, 2014). For *Encephalitozoon* spp, replication occurs within a parasitophorous vacuole into the cytoplasm of the host cell by both binary and multiple fission. It culminates by spore production (sporogony), which represents the mature and infectious form of the parasite. The spore is bound by a thick chitin-rich wall and is released into the external environment following host’s cell wall rupture (Couzinet et al., 2000; Franzen and Müller, 2001; Texier et al., 2010). Both innate and adaptive immune responses are essential for responses against microsporidian infections, but not effective to completely eliminating parasites. *E. cuniculi* parasites are indeed described as hackers of host innate immune defenses (Dalboni et al., 2021). They escape Mo immunity by inhibiting phagolysosomal fusion or by germinating directly into the cytoplasm of these cells (Franzen and Müller, 2001; Aseeja et al., 2021). They also favor efferocytosis and polarize Mo to an M2 profile, facilitating their survival and multiplication inside these cells (Pereira et al., 2019; Dalboni et al., 2021). Polymorphonuclears (PMN) are other key innate immune cells of the first line of defense during microsporidian infections, but no study describes the nature of their interactions with microsporidian spores. Most of the few studies on the modulation of host immune responses by Microsporidia have been performed *in vitro* and the analysis of the dynamic processes at the cellular level in tissues inoculated with *E. cuniculi* parasites is still an unexplored field. The mouse ear pinna infection model could prove useful in overcoming these challenges as the ear pinna is a thin and accessible tissue that is widely used to perform intravital confocal live imaging. The high concentration of Langerhans cells in the epidermis and Mo in the dermis allows the analysis of immune cell behavior in an inflammatory biological context (Amino et al., 2006; Peters et al., 2008; Forestier et al., 2017; Abdul Hamid et al., 2020). In the present study, using the transgenic fluorescent reporter laboratory mice line LysM-EGFP, the mice ear pinna model allowed us to analyze the dynamics of recruitment of enhanced green fluorescent protein (EGFP) expressing phagocytes, in particular PMN and Mo, by intravital imaging.

After loading *E. cuniculi* Calcofluor White (CW) fluorescent spores into the ear pinna dermis, our first results showed that the inflammatory responses to Microsporidia can be analyzed and quantified at both tissular and cellular scales. Parasites induced a rapid and massive inflammatory response at the injection sites, with different cellular dynamics and motility parameters (average speed, displacement length, straightness of the trajectory) of recruited cells. These cells interacted with spores at the injection site, leading to different parasite fates: clearance or maintenance. Finally, the ability of *E. cuniculi* parasites to proliferate in the ear pinna was evidenced. We therefore developed a mice model of parasite infection to analyze the dynamics of inflammatory responses against Microsporidia at the tissue and cellular levels. Our first results show that a way for parasite to persist in its host is to hack phagocytes.

## 2. Materials and methods

### 2.1. Mice and ethics statement

LysM-EGFP transgenic mice (10- to 12-week-old males and females) were obtained from the Biology and Genetics of Bacterial Cell Wall unit, Pasteur Institute (Paris, France) and bred in the animal care facility at Université Clermont Auvergne (Clermont-Ferrand, France). Infected mice were housed in ventilated plastic cages. All experiments were approved by Ethics Committee on Animal Experimentation of Auvergne (C2E2A), Clermont-Ferrand, France (agreement number: 28868) and carried out in accordance with the applicable guidelines and regulations.

### 2.2. Development of *Encephalitozoon cuniculi* spore preparations

*Encephalitozoon cuniculi* (GB-M, genotype I) spores were propagated in HFF (Human Foreskin Fibroblast) cells maintained at 37°C, 5% CO_2_ in MEM supplemented with 10% SVF and 1% L-Glutamine. Spores were harvested from the supernatant of infected cells, washed twice and resuspended in PBS after two successive centrifugations (5 min at 5000 rpm). The numbers of HFF residual cells and/or cellular debris in the spore preparation was evaluated after staining in a 0.025% Trypan Blue solution (Gibco™). Spores were stored in PBS at 37°C, 5% CO_2_. For all experiments, “freshly prepared” spores were used, i.e., collected within previous 8 days.

### 2.3. Fluorescent labeling of *Encephalitozoon cuniculi* spores

Purified *E. cuniculi* spores were stained with a 0.1% Calcofluor White M2R solution (Sigma Aldrich) in PBS at room temperature for 2 min. After a wash in ultra-pure water, spores were centrifuged for 5 min at 1000 rpm, resuspended in PBS and immediately used for infection assays.

### 2.4. Scanning electron microscopy observation of purified *Encephalitozoon cuniculi* spores

Ten million *E. cuniculi* CW-labeled spores were deposited on 0,2 μm SEM Pores (DTM9305, Jeol filters) with a 34G needle fitted to a NanoFil Syringe. After absorption, spores were fixed for 12 h at 4°C in 0.2 M/sodium cacodylate buffer that contained 4% paraformaldehyde and 2.5% glutaraldehyde, at pH 7.4. They were then rinsed for 30 min in the same buffer and post-fixated for 1 h at room temperature (RT) with 1% osmium tetroxide in sodium cacodylate buffer 0.2 mol/L, at pH 7.4, that contained 4% paraformaldehyde and 2.5% glutaraldehyde. Samples were then washed 30 min in sodium cacodylate buffer (0.2 mol/L, pH 7.4) and post-fixed 1 h with 1% osmium tetroxide in the same buffer. After washing for 20 min in distilled water, dehydration by graded ethanol was performed from 25 to 100° (10 min each) to finish in hexamethyldisilazane (HMDS) for 10 min. Filters were mounted on stubs using adhesive carbon tabs, coated with carbon (Quorum Q150 TES Plus) and analyzed with the scanning electron microscope Hitachi Regulus 8230 (Japan) at 2 kV. Images were obtained using the secondary electrons detector.

### 2.5. *Encephalitozoon cuniculi* spores infectivity assay

#### 2.5.1. *In vitro* cell infection

Human Foreskin Fibroblast cells were grown at 37°C, 5% CO_2_ in MEM supplemented with 10% SVF and 1% L-glutamine. Cells were distributed on coverslips in 24 well cell culture plates (8 × 10^4^ cells/well) and incubated overnight. Purified labeled *E. cuniculi* spores were then added to cell cultures (50 spores for 1 host cell). After 1 h of contact, cells were washed with MEM and maintained in supplemented media. Three to 6 days post-infection (pi), coverslips of infected cells were fixed using Farmer’s fixative (1/4 glacial acetic acid, 3/4 absolute ethanol) for 2 h at 4°C and stored in absolute ethanol at 4°C.

#### 2.5.2. Quantification of infectious foci

Infectious foci were observed using fluorescence *in situ* hybridization staining (FISH) method (Dubuffet et al., 2013), followed by DAPI and Direct Yellow (DY96) counter-staining. Using a probe designed here to target specifically *E. cuniculi* rRNA (Ec01, 5′-CCACAGGGGCAGACCACTAT-3′), FISH labeling allowed detection of all Microsporidia stages, except spores. The combination of DAPI (nucleic acid) and DY96 (chitin) staining enabled the visualization of infection foci, regardless of the parasite stages they contain (meronts/sporoplasm: FISH; sporonts/sporoblasts: FISH and DY96; spores: DY96). Coverslips of infected cells were subsequently rehydrated in PBS at RT for 15 min, then incubated in a 1:1 solution of PBS and hybridization buffer (HB: 20 mM Tris–HCl pH 7.8, 0.9 M NaCl, 1X Denhardt’s solution, 0,01% SDS), to finish in HB at 47°C for 20 min. The 5′-Cy3.5-labeled probe was then added at a concentration of 0.5 μM and hybridization was realized at 47°C for 2 h. Samples were then washed in HB at 47°C for 30 min, then in HB:1X PBS at RT for 30 min and finally in 1X PBS at RT. Samples were counter-stained using 100 μg/L DAPI, 1 μM DY96 and 0,1% SDS at RT overnight. Coverslips were mounted in Prolong Diamond (Thermo Fisher Scientific) and observed with 63X (oil) objective on ZEISS Axio Imager microscope (Carl Zeiss Microscopy, Germany). Acquisition was performed with 3 excitation/emission filters to observe fluorescence emitted from the FISH probe (ex: 590 nm; em: 610 nm), DAPI (ex: 350 nm; em: 470 nm) and DY96 (ex: 391 nm; em: 491 nm). The entire surface of the coverslip was reconstructed using Zen 3.3 software and the number of infection foci in ratio to the total number of cells was counted on 70% of the coverslip surface. The counting of infection foci was carried out via the Imaris software using the spot tool. One spot was equivalent to one infectious focus observed in the channel corresponding to the FISH probe, with a minimal average diameter of 10 μm.

### 2.6. Inoculation of fluorescent *E. cuniculi* spores into the ear dermis of mice

Mice were anesthetized by intraperitoneal injection of a mixture of ketamine (50 mg/kg) and xylazine (5 mg/kg). Ten million CW labeled spores were microinjected in a small volume (2 μL per injection site, 4 μL in total) into the dorsal ear dermis of anesthetized mice using a 34G needle fitted to a NanoFil syringe (World Precision Instruments). Control mice were inoculated with the same volume of PBS. A characteristic papule, evidence of an intradermal injection, was observable at the 2 microinjection sites.

### 2.7. *In vivo* confocal intravital imaging: acquisition

#### 2.7.1. Real time video acquisition

Two to 72 h pi or post-inoculation of PBS, mice were again anesthetized, and ears were laid flat on a coverslip and imaged on a ZEISS Cell Observer Spinning Disk confocal microscope (Carl Zeiss Microscopy, Germany), as previously described (Abdul Hamid et al., 2020). Briefly, video acquisition was performed with 2 different lasers to observe both EGFP and CW fluorescence (ex: 488 nm and 347 nm; em: 509 and 432 nm, respectively) with exposure time set at 100 ms. Acquisition was carried out with 10X (dry), 20X (dry) and 40X (oil) objectives for 15 to 20 min periods. With the 10X objective, multiple fields of observation were acquired in order to image the entire injection site. Acquisition was repeated at 24, 48, and 72 h pi.

#### 2.7.2. Mosaic acquisition

Ear tissues of both infected and control mice were imaged from 4 to 7 h to 10 days pi on a ZEISS LSM 800 confocal microscope (Carl Zeiss Microscopy, Germany), as previously described (Abdul Hamid et al., 2020). EGFP and CW signals were acquired with an exposure time of 15 and 30 ms, respectively. Each imaging session lasted 30 to 45 min in order to get a reconstruction of the entire tissue.

### 2.8. *In vivo* confocal intravital imaging: data analysis

#### 2.8.1. Image processing and video analysis

Videos acquired with the 10X objective were processed using ZEN 3.3 software. They were first stitched together, and certain plans and time points were further chosen to follow the inflammatory response and host-parasite interactions over time. Videos acquired with the 20X objective were analyzed using the “Spots” tool on Imaris software. A track was generated for each cell and trajectories of joining cells were removed manually, as previously described (Abdul Hamid et al., 2020). Three parameters of EGFP + cells displacement properties were extracted for each time point: the average speed, the straightness and the displacement length.

#### 2.8.2. Mosaic analysis

Reconstructed images acquired on the ZEISS LSM 800 confocal microscope were stitched together using ZEN 3.3 software to reconstitute an entire image of the ear tissue at each time point. A maximum intensity projection was created from the different image Z-stacks. A ROI was drawn manually around the EGFP fluorescence zone of the 24 h pi image. The shape of this ROI was then applied to the other time points images, as described previously (Abdul Hamid et al., 2020). An ROI was also drawn around the CW fluorescence zone at the early time point (2 to 6 h pi) and then applied to the other time points images. The ratio of the sum of intensities of EGFP or CW fluorescence to the area of the ROI was then calculated for each time point.

### 2.9. Immunolabelings

Ear pinna tissues were collected after sacrifice of infected and control mice, from 2 h to 7 days pi or post-inoculation of PBS. Tissues were then quickly frozen on dry ice using Optimal Cutting Temperature Compound (OCT) embedding medium to allow tissue preservation. Ten μm-thick ear cryosections were prepared on a per cryostat (CM 1950 Leica), with a frequency of recovery of one cut every 50 μm until the block was exhausted (CICS platform, Faculty of Pharmacy, UCA). Cryosections were further stored in a freezer at −80°C. To perform immunolabelings, cryosections were slightly defrosted at RT for 30 min, rehydrated with PBS for 30 min again, and then incubated with a PBS-Triton 0.1%-BSA 2% solution for 1 h. Specific antibodies were further applied for 3 h at RT or overnight at 4°C: an anti-keratin antibody (Cytokeratin 5-coupled to AlexaFluor 546, 20 μg/mL, Santa Cruz Biotechnology, Inc.) to visualize epidermis and a polyclonal antiserum obtained from an *E. cuniculi* infected rabbit (2 μg/mL) coupled with an anti-IgG-AlexaFluor 647 labeled secondary antibody (2 μg/mL). The anti-*E. cuniculi* polyclonal antibodies identify all parasitic development stages (data not shown). All samples were counter-stained using 100 μg/L DAPI and mounted with ProLong™ Diamond Antifade Mountant (Thermo Fisher Scientific). Images were acquired on ZEISS LSM 800 confocal microscope (Carl Zeiss Microscopy, Germany) and observed with 20X and 40X objectives (CLIC platform, Faculty of Pharmacy, UCA). Three different lasers were used to observe the CW fluorescent signal of *E. cuniculi* spores, along with the DAPI signal of both cell and parasite nuclei (ex: 405; em: 410–546), the EGFP signal of phagocytes (ex: 488; em: 410–546), the AlexaFluor546 labeled anti-Keratin antibody specific signal of epidermis layer (ex: 561; em: 555–620) and the signal of the AlexaFluor647 coupled with the anti-*E. cuniculi* antibody to detect all parasitic stages (ex: 640; em: 655–700).

### 2.10. Statistical analysis

Data generated were analyzed using a Mann-Whitney non-parametric two tailed test on Prism 5 software (GraphPad Software, Inc). *p* ≤ 0.05 was considered statistically significant (symbols: ^***^p < = 0,001; ^**^p < = 0,01; * < = 0,05; ns = non-significant).

## 3. Results

### 3.1. Preparation and characterization of calibrated inocula of *Encephalitozoon cuniculi* spores for inoculation into the mouse ear pinna

**FIGURE 1:**
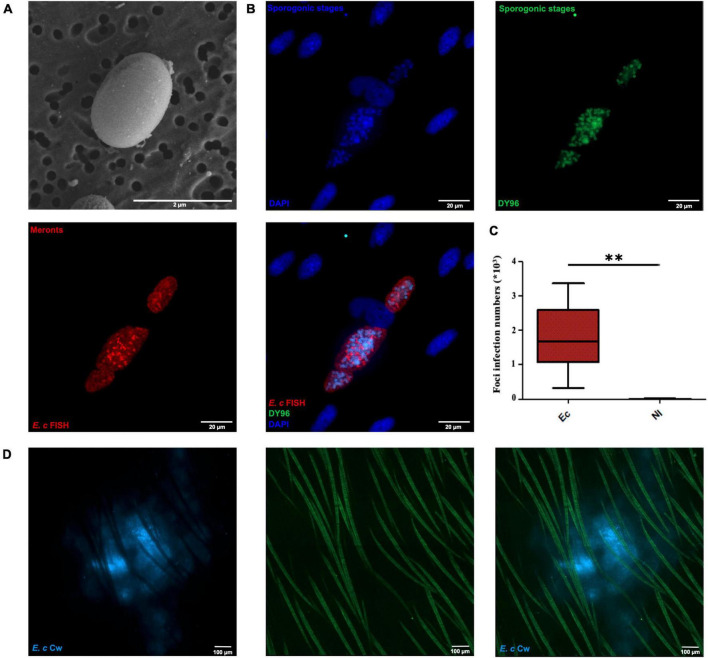
Properties of calibrated inocula of *Encephalitozoon cuniculi* Calcofluor White labeled spores. SEM micrograph of a CW-labeled spore **(A)**. Fluorescence microscopy images of a *E. cuniculi* infectious foci inside HFF cells after FISH labeling and DY96 counterstaining **(B)**. *E. cuniculi* foci infection numbers inside HFF cells 3 days pi. **: Statistically significant difference compared with the control group (PBS) with *p* < 0.005. Mann-Whitney statistical test. Five independent experiments, *n* = 15 *Ec* infected cells and *n* = 15 non-infected (NI) cells **(C)**. Reconstituted confocal images of the intradermal injection site showing the maximal projection intensities of the CW signal. Autofluorescent hairs appear in green **(D)**. Scale bar: 10 μm.

A reproducible protocol of *E. cuniculi* spore preparation was set up to obtain calibrated inocula of 10^7^ spores in 4 μl (total injection volume). As *E. cuniculi* is an intracellular parasite, many subcultures of HFF were required to obtain large quantities of spores for all experiments. Spores were collected from the supernatants of infected HFF cell cultures and were labeled with CW, a vital fluorescent dye that stains chitin. The proportion of cellular debris in the spore suspension was very low, equal to 0.20 ± 0,02% (*n* = 5 independent experiments). In parallel, the quality of the spore suspension was evaluated by performing immunolabelings using a polyclonal anti-*E. cuniculi* (*Ec*) serum. As illustrated on Supplementary Figure 1A, the majority of parasites were co-labeled with both CW dye (CW +) and polyclonal antibody (*Ec* +) and corresponded to sporoblast and spore stages. The small proportion of CW-*Ec* + parasites (filled white arrows) revealed the presence of other parasitic stages in the inoculum, but in a minority. Scanning electron microscopy (SEM) analysis showed that *E. cuniculi* spores have a characteristic ellipsoid (Figure 1A) or more rounded (Supplementary Figure 1B) morphology, with a smooth and homogeneous surface. Evaluation of inoculum infectivity *in vitro* by FISH labeling and DY96 counterstaining revealed the presence of infectious foci inside HFF cells after 72 h. Meront stages present at the periphery of the foci were labeled by FISH whereas sporogonic stages (sporonts, sporoblasts, and/or spores) were labeled by DY96 in the center (Figure 1B). Quantification of the infectious foci numbers revealed very high numbers in presence of inoculum, as compared to uninfected cell condition (Figure 1C), indicating that CW labeling and spore treatments during inoculum preparation permits them to stay infectious. Finally, LysM-EGFP transgenic mice were inoculated intradermally into the ear pinna with 10^7^ spores. Image acquisitions on confocal Spinning Disk at low magnification (x10) allowed us to visualize the inoculum in the cutaneous tissue, on the surface and in depth, as illustrated on Figure 1D.

### 3.2. Micro-injection of calibrated inocula of *Encephalitozoon cuniculi* fluorescent spores into the mouse ear pinna: monitoring the inflammatory responses and the fate of the inocula

**FIGURE 2:**
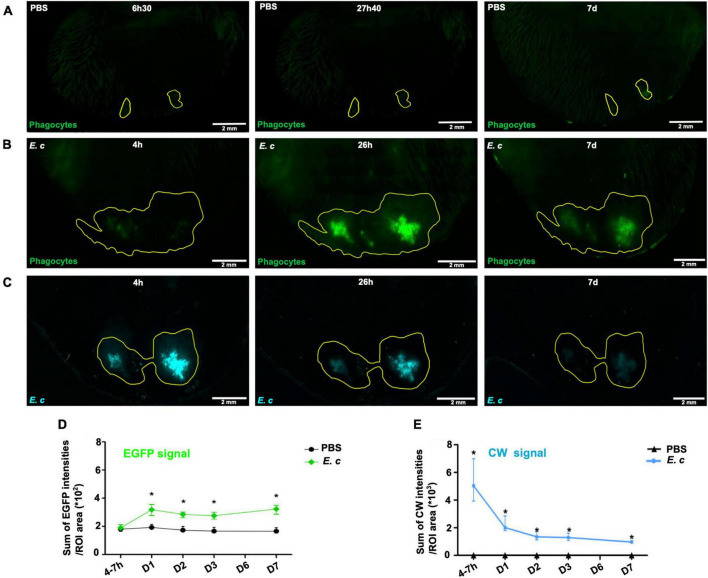
Inflammatory responses after micro-injection of calibrated inocula of *Encephalitozoon cuniculi* spores in the mouse ear pinna. **(A–C)** Reconstituted confocal images of the mouse ear pinna tissue showing the maximal projection intensities of the EGFP and CW signals. LysM-EGFP transgenic mice were microinjected with PBS **(A)** or CW-labeled *E. cuniculi* spores (*E. c*) **(B,C)** at early (4–6 h30) and later (after 24 h pi) time points. The EGFP fluorescence (green) signal corresponds to phagocytes (PMN and Mo). An ROI was defined around the phagocyte recruitment area 26 h pi or 27 h 40 post-injection (PBS) (yellow line) where the “Sum of EGFP fluorescence intensities” was measured. The same ROI was then applied to the images of the other kinetic points **(A,B)**. The CW fluorescence (blue) signal corresponds to *E. c* spores. An ROI was defined around the inoculum area 4 h pi (yellow line) where the “Sum of CW fluorescence intensities” was measured. The same ROI was then applied to the images of the other time points **(C)**. One representative experiment is shown for each group of mice from 2 independent experiments. Scale bar: 2 mm. Ratios of the sum of EFGP **(D)** or CW **(E)** fluorescence intensities to ROI area for infected (*E.c*) or control (PBS) mice. *: statistically significant difference compared with the control group (PBS) with *p* < 0.05. Mann-Whitney statistical test. Four independent experiments, *n* = 8 tissues (*E. c*) and *n* = 14 control tissues (PBS).

The inflammatory responses and the fate of the inocula were followed in the ear tissue of LysM-EGFP mice inoculated with 10^7^ *E. cuniculi* fluorescent spores or with PBS (control mice). Macroscopically, signs of an inflammatory response were detectable from 24 h pi, with redness and swelling at the injection site, as well as a slight vasodilation and punctiform hemorrhagic areas persisting until 7 days pi on inoculated ear tissues (Supplementary Figure 2). Using a confocal microscopy approach, inflammatory responses and parasite fate were followed at early (4–7 h pi) and later time-points (from 24 h to 7 days pi) by measuring the intensity of the EGFP (phagocytes) and CW (spores) signals for each group (Figure 2). Reconstruction of the entire surface of the ear pinna tissue by imaging allowed us to follow the global inflammatory responses against *E. cuniculi* spores and the fate of the inocula over time in the entire tissue. The EGFP and the CW signals were quantified at the injection sites inside defined regions of interest (ROI), using a previously set up protocol (Abdul Hamid et al., 2020). For infected mice, a marked inflammatory response was observed in the atrial tissue at 24 h pi (Figure 2B), as compared to uninfected control mice (Figure 2A). Plotting the sum of fluorescence intensities relative to the tissue area defined by the ROIs for the different time points showed a twofold increase of the EGFP signal at this time-point (Figure 2D). The signal intensity further decreased to finally stabilize from day 3 pi. Interestingly, values remained 1.5-fold higher than values of control group until 7 days pi, illustrating a persistent inflammatory response in the infected condition (Figure 2C). Parasite load was also monitored via analysis of the intensity of the CW signal emitted by the spores into the ear tissue over time (Figures 2C, E). A decrease of inoculum fluorescent signal was observed between the early time points and 24 h pi. However, this signal was still detectable at the inoculation site up to 7 days pi, illustrating some spore maintenance in the cutaneous tissue, at least within this time period (Figure 2E).

### 3.3. Different phagocytes dynamics and parasite fates early after the microinjection of *Encephalitozoon cuniculi* spores into the mouse ear pinna

**FIGURE 3:**
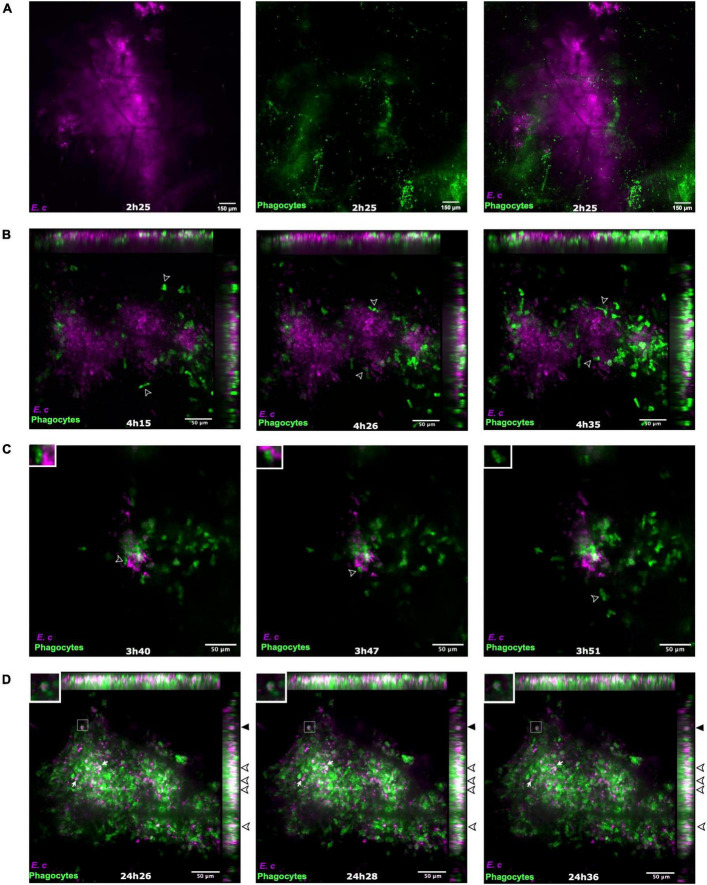
Dynamics of recruited EGFP + cells in the mouse ear pinna after inoculation of *Encephalitozoon cuniculi* spores. Live confocal imaging after micro-injection of *E. cuniculi* spores in the ear pinna of LysM-EGFP transgenic mice from 2 h to 25 h pi **(A–D)**. The recruited EGFP + phagocytic cells progressively infiltrate the injection area **(A)** and come into contact with CW + parasites at the injection site [**(B)**, empty arrowheads follow the same cell over the course of the acquisition]. Different cell behaviors are observed, an “arrested” **(B)** and a “quick” phenotype. The “quick” phenotype corresponds to cells that interact shortly with parasites and rapidly leave the injection area [**(C)** empty arrowhead and inset]. Live confocal imaging at later time points after micro-injection of *E. cuniculi* spores **(D)**. White arrows and empty arrowheads indicate the presence of numerous remaining parasites after 24 h. Inset and filled black arrowheads show the decrease of the CW fluorescence signal inside an EGFP + phagocyte during the video acquisition time, in favor of parasite phagocytosis and lysis inside the cell **(D)**. **(A–D)** Images show average intensity projections of green (phagocytes) and blue (parasites) fluorescence. Some intracellular parasites appear in white [overlap of green and blue fluorescent signals, empty arrowheads, **(D)**]. 4 independent experiments, *n* = 8 tissues (*E. c*) and *n* = 14 control tissues (PBS). Scale bar: 50 μm.

LysM-EGFP transgenic mice were inoculated intradermally into the ear pinna with 10^7^ *E. cuniculi* spores or with PBS (control mice). Videos acquisitions (between 15 and 20 min) were performed at both injection sites to analyze in real time the dynamics of mobilization of inflammatory cells at early time points (2–4 h) and 24 h pi. As early as 2 h pi, EGFP + phagocytic cells were massively mobilized at the injection sites in mice inoculated with parasites (Figure 3A). In control mice, a lower cell recruitment was detected 24 h pi, due to trauma caused by the needle during microinjection (Supplementary Figure 3A and Supplementary Video 1). In the presence of spores, recruited cells were first localized at the periphery of the injection area, to finally invade it and interact with parasites (empty arrowheads) (Figure 3B, Supplementary Figure 3B and Supplementary Video 2). Different cell behaviors were thus observed. Some phagocytes rapidly left the injection sites after a quick interaction with parasites or without interacting with spores (Figure 3C, empty arrowhead and inset, and Supplementary Video 3). At 24 h pi, most of the recruited cells arrested at the injection site, leading to a decrease of the CW fluorescent parasite signal due to parasite lysis during the video acquisition time (Figure 3D, inset and black filled arrowheads). Other intracellular parasites were detected inside EGFP + cells, with the maintenance of a bright CW + fluorescent signal during the video acquisition time (Figure 3D, filled white arrows and arrowheads and Supplementary Video 4). This observation highlights the absence of parasite degradation and its potential maintenance inside phagocytes.

### 3.4. Monitoring of inflammatory responses into the mouse ear pinna: partial ineffectiveness to eliminate *Encephalitozoon cuniculi* parasites over time

**FIGURE 4:**
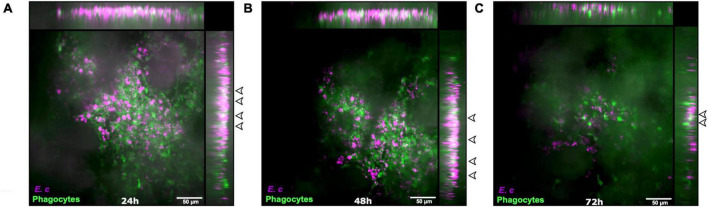
Parasite maintenance in the mouse ear pinna after inoculation of *Encephalitozoon cuniculi* spores. Live confocal imaging after micro-injection of *E. cuniculi* spores in the ear pinna of LysM-EGFP transgenic mice 24 h **(A)**, 48 h **(B)**, and 72 h **(C)** pi. Images show average intensity projections of green (phagocytes) and blue (parasites) fluorescence. Some intracellular parasites appear in white (overlap of green and blue fluorescent signals, empty arrowheads). Cluster of cells interacting with spores are observed at all-time points. Empty arrowheads indicate the presence of remaining parasites from 24 h to 72 h pi. Four independent experiments, *n* = 8 tissues (*E. c*) and *n* = 14 control tissues (PBS). Scale bar: 50 μm.

LysM-EGFP transgenic mice were inoculated intradermally into the ear pinna with 10^7^ *E. cuniculi* spores or with PBS (control mice). The dynamics of phagocytes recruitment and the parasite fate were followed in real time and over the course of infection at both injection sites, from 24 to 72 h pi. A massive clustering of recruited EGFP + phagocytes was observed at 24 h pi (Figure 4A). The number of cells decreased significantly at day 3, probably due to PMN apoptotic death and clearance (Figure 4C). Numerous parasites were still present in cell recruitment areas over time, with some bright fluorescent CW + intracellular parasites detected, even at 72 h pi (Figures 4A–C, empty arrowheads). These observations highlight (i) the *in vivo* maintenance of some parasites inside phagocytes, and (ii) the partial ineffectiveness of inflammatory responses to clear parasite infection within 72 h.

### 3.5. Motility properties of recruited innate immune cells are modified in presence of *Encephalitozoon cuniculi* spores into the mouse ear pinna

**FIGURE 5:**
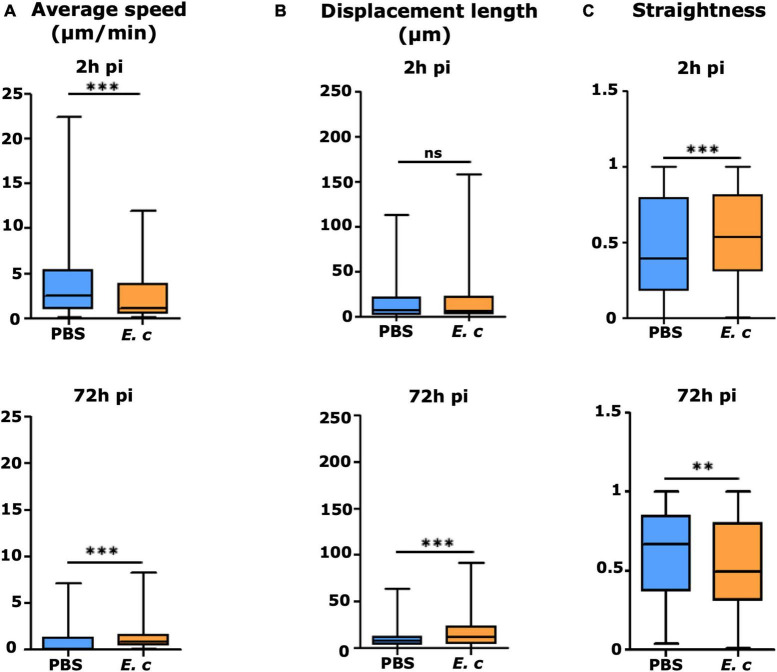
Displacement properties of recruited EGFP + cells in the mouse ear pinna after inoculation of *Encephalitozoon cuniculi* spores. Average speed **(A)**, displacement length **(B)**, and straightness of trajectory **(C)** of EGFP + recruited cells to injection sites at early (2 h pi) and late (72 h pi) time points after inoculation of *E. cuniculi* spores or PBS (control) in the ear pinna tissue of LysM-EGFP mice. Data are expressed as median and interquartile ranges pooled from 2 (*E.c*) or 6 (PBS) different mice in 3 independent experiments. Number of cells (N) analyzed for each group at early and late time points, respectively, Control: *N* = 496 and 248 cells; Infected: *N* = 394 and 946 cells. Statistically significant difference compared to PBS control for the 3 displacement parameters with ^**^p < 0.005 and ^***^p < 0.0001.

Using Imaris software, the motility properties (average speed, straightness, displacement length) of recruited cells were analyzed from the previously acquired time-lapse videos. A previously set up analysis protocol was used, and cell trajectories were analyzed after 2 h (early time-point) and 72 h (later time-point) (Abdul Hamid et al., 2020; Figure 5). The motility of all cells at the injection areas were analyzed in response to *E. cuniculi* spores or to PBS (control mice). In infected mice, both cells that interacted with parasites or cells that did not were included. The presence of parasites modified the 3 motility parameters of recruited cells, as compared to the control condition (Figure 5). At 2 h pi, average speed was significantly decreased (Figure 5A), indicating that most of the recruited cells arrest at the injection site to interact with inoculated spores. The displacement length was, however, comparable for the 2 groups of mice (Figure 5B). Interestingly, an opposite effect was observed for the straightness parameter, with a significant increase in the presence of parasites (Figure 5C). This indicates that behaviors of recruited cells are heterogeneous at early time-point in presence of parasites. At 72 h pi, the straightness parameters were significantly decreased in the presence of parasites, at the opposite of the average speed and displacement length parameters which were increased in the presence of parasites. These results indicate that parasite infection maintenance in the cutaneous tissue impacts cell motility parameters.

### 3.6. *Encephalitozoon cuniculi* microsporidia have the ability to proliferate into the mouse ear pinna

**FIGURE 6:**
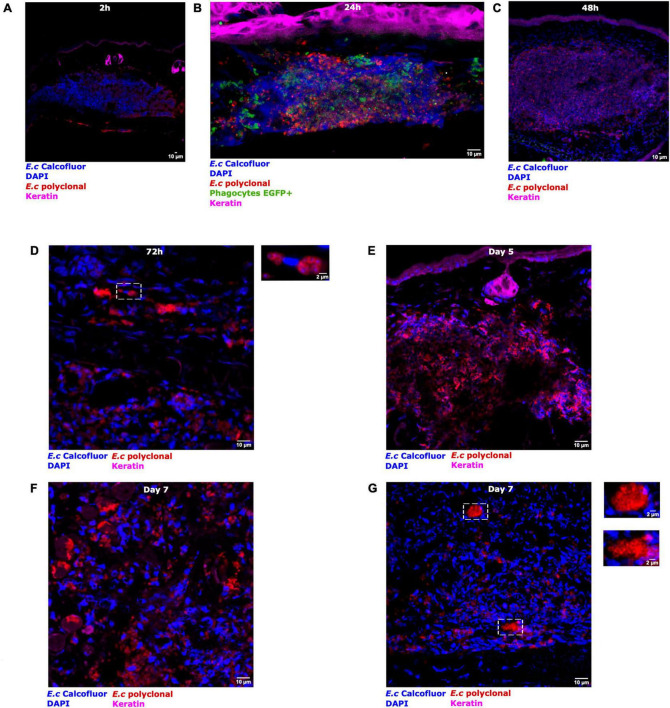
Parasite development after micro-injection of *Encephalitozoon cuniculi* spores in the mouse ear pinna. Confocal immunofluorescence images of immunolabeling cryosections of infected ear tissues. LysM-EGFP mice (EGFP + phagocyte, green fluorescence signal) were inoculated with fluorescent *E. cuniculi* spores (CW + , blue fluorescence signal). Immunostaining was performed on cryosections obtained from 2 h to 7 days pi **(A–G)**. Magenta, red, and blue fluorescence signals correspond to specific labeling of keratin (K5 mAb), all parasite stages (anti-*E. cuniculi* polyclonal serum), and cell nuclei (DAPI)/spores (CW), respectively. A recruitment of EGFP + phagocytes is visualized in the injection area, around the spores inoculum 24 h pi **(B)**. Insets show infectious foci of developing parasites at 72 h [**(D)** in close proximity to cell nucleus] and at day 7 pi **(G)**. [DAPI staining **(G)**]. Three independent experiments, *n* = 30 tissues (*Ec*) and *n* = 12 tissues (PBS). Scale bar: 10 or 2 μm (insets).

**FIGURE 7:**
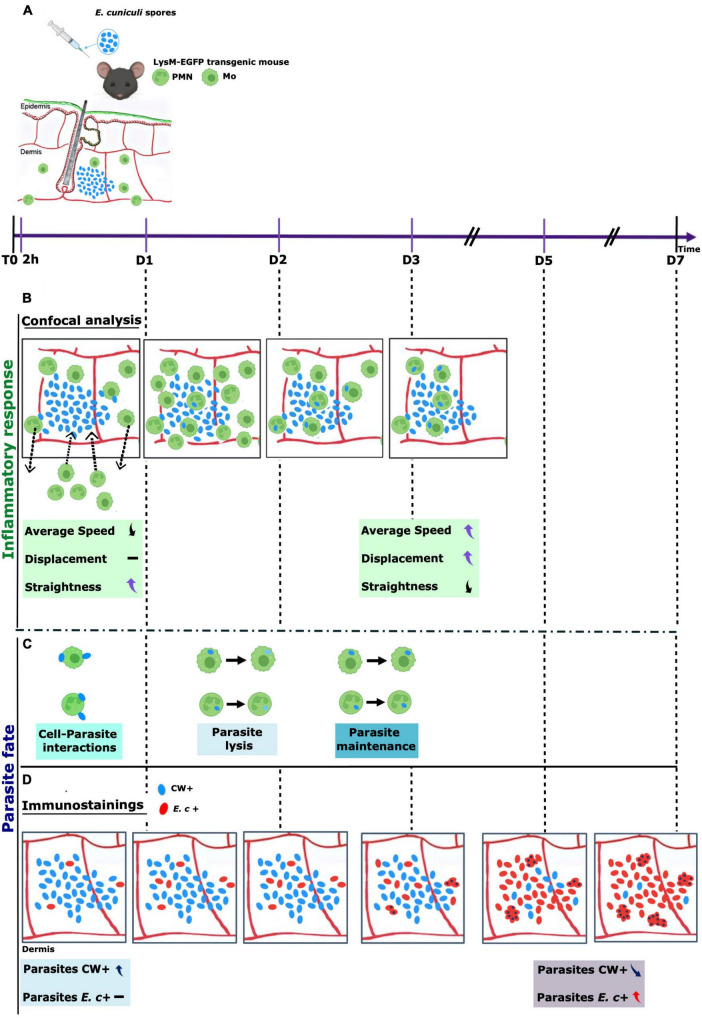
Timeline of *E. cuniculi* infection in the mouse ear pinna model. Calcofluor White (CW + , blue) labeled spores of *Encephalitozoon cuniculi* were inoculated intradermally into the ear pinna of LySM-EGFP mice (EGFP + phagocytes, green). Fluorescent resident Mo, and circulating monocytes and PMN are detected in the mouse ear pinna tissue **(A)**. The presence of the parasite in the tissue induced a massive (peak at 24 h pi) and persistent inflammatory response over time (until day 7 pi). Phagocytes were recruited to the injection site as early as 2 h pi (incoming arrows). Different cell behaviors were observed. Some cells decreased their speed and sometimes arrested to interact with spores. Other cells left rapidly the injection site after interacting or not with parasites (outcoming arrows). Cell motility parameters of the recruited cells were measured at 2 h pi and 72 h pi: speed average, displacement length and straightness were modified in presence of parasites. At 2 h pi, average speed was decreased whereas straightness was increased. At 72 h pi, average speed and displacement length were increased whereas straightness was decreased **(B)**. Numerous spores were observed inside EGFP + phagocytes from 24 h to 72 h pi. For some parasites, the CW fluorescent signal decreased during the video acquisition time, in favor of parasite phagocytosis and lysis inside phagocytes. For other parasites, the CW fluorescent signal continued, in favor of parasite maintenance inside phagocytes **(C)**. Immunolabellings were performed on ear tissue cryosections of infected mice, from 2 h to 7 days pi. CW + : spores, blue; *E.c* + : other parasite development stages, red. Increasing numbers of *E.c* + parasites were detected over time, with the presence of high numbers of infectious foci at days 5 and 7 pi in the dermis. These observations were strongly in favor of the ability of *E. cuniculi* to develop and to proliferate in the mouse ear pinna model showed higher an increase of the *E. c* + signal and a decrease in the Cw + signal over time. The formation of infectious foci as early as 5 days and which grew over time These observations are in favor of the development of the parasite in the tissue and the partial efficiency of phagocytosis **(D)**.

To assess if parasites could develop into the skin, immunolabelings were performed on cryosections of infected ear tissues from 2 h to 7 days pi (Figure 6). At 2 h pi, the majority of parasites of the injection site were double positive CW + *E.c* + (Figure 6A). Recruited EGFP + phagocytes were detected in the dermis, in contact with *E. cuniculi* spores of the inoculum (Figure 6B). Over time, the proportion of CW + *E.c* + parasites decreased, while the proportion of mono-labeled *E.c* + parasites increased, mainly at days 5 and 7 pi (Figures 6B–G and Supplementary Figures 4A–D). In particular, clusters of CW-/*E.c* + parasites were observed at day 7 pi, in favor of infectious foci containing other developmental parasite stages (meronts, sporonts) than spores of the initial inoculum (Figure 6G and Supplementary Figure 4D). Insets of the Figure 6D and Supplementary Figure 4D show foci of developing parasites in close proximity to cell nucleus (DAPI staining), in favor of the presence of developing intracellular parasites, as observed *in vitro* inside HFF cells (Supplementary Figure 4E). These qualitative results were in favor of the ability of *E. cuniculi* parasites to develop in the ear pinna tissue and thus to cycle *in vivo*. The newly set up model will therefore allow us to follow the overall immune response during the microsporidia infection (Figure 7). The main difficulties and facilities of using this model to study immunity against Microsporidia are summarized in Table 1.

**TABLE 1:** Critical points of the mouse ear skin model to study the dynamics of immune responses against *Encephalitozoon cuniculi* parasites^1^.

	Strengths	Limitations
Inoculum preparation and injection	Limits invasive actions (such as biopsies) Possibility to work on a sleeping anesthetized animal Small number of animals required	Needs to label the spores beforehand to see them fluorescent (spore wall labeling with CW dye) Expertise required for micro-injections Injection of a small volume of inoculum to limit non-specific inflammation
Video acquisition	Confocal microscope allows to make observations up to 150 μm of the cutaneous tissue surface The mouse ear pinna is easy to image by confocal microscopy (accessible tissue) 3D acquisition Real-time imaging during long periods of time Parasites present in the microscopic fields of view can be visualized	Deep anesthesia required. Otherwise, risk of movement of the animal (more anesthetic can be injected during recording if necessary) Monitor the animal and use equipment to maintain a normal body temperature (warming blanket) Manipulate the ear pinna tissue very gently Video acquisition limited by the microscopic field
Video analysis	Visualization of the dynamics of interactions between immune cells and parasites Fate of parasites present in the microscopic fields of view can be monitored over time Intracellular parasites can be visualized inside EGFP + phagocytes The behavior of recruited phagocytic cells can be analyzed Cell motility parameters of recruited cells can be analyzed	Autofluorescence of the epidermis and of hair follicles Persistence of the fluorescent CW + signal does not reflect the parasite development Saturation of the EGFP + signal induced by the inflammatory response at 24 h pi (peak) Knowledge of tracking software required Analysis of high numbers of cells required for tracking analysis

^1^Amino et al. (2007) and Beignon et al. (2014).

## 4. Discussion

The dynamics of the implementation of innate immune responses during microsporidian infection *in vivo* is a determining factor for parasite fate. As the first line of defense, these responses mostly involve inflammatory cells such as PMN, Mo and dendritic cells, and allow partial elimination of parasites (Dalboni et al., 2021). Importantly, they condition the nature of the adaptive immune responses that will be set up during host infection. Microsporidia are capable of establishing a persistent infection in their host by evading host immune responses. As an example, and based on *in vitro* observations, *E. cuniculi* parasites can hack phagocytes such as Mo (Han et al., 2020). *In vivo*, few studies explored the dynamics of interactions between inflammatory cells and Microsporidia in mammalian hosts. The parasite immune evasion mechanisms and their chronology are thus currently difficult to understand and to follow over time. In the present study, we analyzed for the first time the dynamics of early innate immune responses to Microsporidia *E. cuniculi* parasites in the skin. This microsporidian species is a model of choice to study immunobiology of these parasites (Khan et al., 2001). It was the first cultivated species, and its genome has been sequenced and annotated (Katinka et al., 2001). It is one of the 17 microsporidian species infecting humans (González-Machorro et al., 2019; Ruan et al., 2021), and the third most common species found in humans, with the ability to disseminate to several organs causing a wide spectrum of pathogenesis including encephalitis, nephritis, hepatitis or keratoconjunctivitis (Ghosh and Weiss, 2012; Dumortier et al., 2021). All Mo hacking observations described *in vitro* used this species (González-Machorro et al., 2019; Dalboni et al., 2021). The mice ear pinna was used as a cutaneous imaging site as it is an accessible and thin tissue that can be rapidly and easily prepared for imaging over long periods of time (Peters et al., 2008; Abdul Hamid et al., 2020). Calibrated inocula of *E. cuniculi* CW + spores were inoculated intradermally into the ear tissue of LysM-EGFP transgenic mice, in a very small volume with a 34G needle to limit non-specific inflammatory responses due to injection trauma.

Standardization of the inoculum was a key point. The first experiments were performed with calibrated inocula of *E. cuniculi* spores collected from the supernatants of infected MDCK cell cultures. The purification of spores required several steps of water washings, centrifugations and filtrations to remove a maximum of cellular debris caused by the detachment of MDCK cells from the cell layers. These multiple treatments altered the morphology and the infectivity of the spores, as a low number of infectious foci were counted *in vitro* on MDCK cells compared to HFF cells infected by the inoculum (data not shown). *In vivo*, the first imaging experiments performed showed heterogeneity in the intensity of the inflammatory responses (EGFP + fluorescent signal) measured in the ear tissue of LysM-EGFP infected mice 24 h pi, probably in relation to the presence of cellular debris still present in the inoculum (data not shown). In order to overcome these drawbacks, the cell lineage used to produce the spores *in vitro* was changed and HFF cells were used. Numerous subcultures were necessary as HFF cells produce a lower number of spores compared to MDCK cells, but they didn’t detach from the cell layers once infected. The number of cells and cellular debris present in the culture supernatants was greatly reduced. By removing the filtration steps and decreasing the number of washes and centrifugations, the infectivity rate of the spores was greatly increased and ultrastructural observations of the spores by SEM revealed typical smooth morphologies. Immunolabelings, using a polyclonal anti-*E. cuniculi* serum, performed on the CW-labeled purified spores revealed the presence of a majority of spores in the inoculum. Nevertheless, a small proportion of other parasitic stages (sporoplasms, meronts and/or sporonts) was detected. These developmental stages could be released in the culture supernatants after lysis of the infected HFF cells. As the spore stage is the only infectious stage capable of maintaining itself in the external environment, the other parasitic stages are most likely rapidly degraded once inoculated into the skin tissue. To obtain spore preparations free of other parasitic stages, a Percoll gradient spore purification method has been described in literature (Dalboni et al., 2021). This method was not used because it could alter the spores and thus decrease their infectivity.

We inoculated a high number of spores (10^7^) into the ear tissue of LysM-EGFP transgenic mice, as in previously published rodent models (Sak and Ditrich, 2005; Kotková et al., 2017; Brdícková et al., 2020; Sak et al., 2021, 2022). Once set up, our protocol enabled us to analyze qualitatively and quantitatively, simultaneously the innate immune responses induced by *E. cuniculi* CW + fluorescent spores in mice and the fate of parasites at the tissue level. Imaging analysis showed that parasite inocula induced an early inflammatory response at the ear pinna injection site, consisting of recruited EGFP + fluorescent phagocytes, in particular PMN and monocytes/Mo. We quantified this response for the first time and observed a significant increase in the numbers of recruited cells at 24 h pi, as well as a persisting response until 7 days pi. These data correlated with the macroscopic observations of inflammatory responses on infected ear tissues, including vasodilation, edema and punctiform redness observed until 7 days pi. The CW + parasite signal was quantified in parallel to the EGFP + phagocytes signal. It decreased drastically after 24 h, which correlates with the massive recruitment of phagocytes at this time-point and the combined phagocytosis and lysis of spores. The parasite signal further slowly decreased but was still present at day 7 pi. These first observations, associated with the ability of the parasites to cycle observed at day 7 pi, are in favor of parasite maintenance in the skin.

The mice ear pinna model then enabled us to follow *in situ* and over time the dynamics of the inflammatory responses to *E. cuniculi* spores at the cellular level. EGFP + fluorescent phagocytes were quickly and massively mobilized from the blood circulation to the inoculation site by diapedesis, probably including monocytes and PMN. Resident Mo were most likely also involved in the inflammatory responses to parasites, as they are present in high concentrations in the ear pinna tissue (Forestier et al., 2017). The behavior of recruited or mobilized phagocytes appeared to be heterogenous, leading to different phenotypes of cell-parasite interactions. At early time-points, bright fluorescent phagocytes, probably PMN, as they are the first cells to be recruited in an inflammatory context (Filep, 2022), crossed the inoculation zone, or slowed down to remain in contact with spores for a very short time before leaving the injection area. These “quick” PMN could leave the injection site in response to chemokine signals and thus participate to parasite dissemination (Nassonova et al., 2001; Sak et al., 2022). Among chemokines that play a role in PMN chemotaxis and activation during microsporidian infection, CXCL5 gene expression is strongly induced in a rodent model (Fischer et al., 2007), but CXCL1 and CXCL8 could also play a key role. Pro-inflammatory and anti-inflammatory cytokines production, including TNFα, IFNγ, IL-6, IL-10, and IL-12, could also be measured in the infected tissues. *Ex vivo* experiments have indeed described the involvement of these cytokines in anti-microsporidian immune responses in mice (Moretto et al., 2010; Bernal et al., 2016; Han et al., 2020). In most cases, early recruited EGFP + cells slowed down or even stopped to interact with spores at the injection site during the video acquisition time. Complementary experiments are required to identify the phenotype of these “slow” vs. “quick” cells, to know if they are PMN or Mo, or different cell subtypes of these phagocytes. In particular, PMN represent a phenotypically heterogenous population of phagocytic cells, with possible distinct functional properties during acute inflammation, depending on the cell subtype (Filep, 2022). The ear pinna model allowed us to describe for the first time *in vivo* the ability of *E. cuniculi* parasites to invade and to maintain inside innate immune cells. Numerous parasites were detected intracellularly few hours pi. They had different fates during the video acquisition time. For some parasites, a decrease of the CW + spores fluorescent signal was observed, in favor of phagocytosis and lysis of the spores. For other parasites, no decrease of the intensity of the CW + signal was observed during the video acquisition time, in favor of the ability of parasites to maintain inside the microbicidal intracellular environment of EGFP + phagocytes. In favor of this hypothesis, Valencáková et al. (2004) described altered functional properties of phagocytes collected from *E. cuniculi* infected mice, with decreased phagocytic activity. Related to this observation, the decrease of the CW + fluorescent signal observed for some spores could also be related to parasite development within phagocytes. In all cases, the *in vivo* maintenance of parasites was responsible for a continuous recruitment of EGFP + phagocytes observed at the tissue level over time (until 7 days pi). Phagocytes harboring intracellular parasites were observed until 3 days pi. According to literature, these phagocytes could travel throughout the host body and display *E. cuniculi* parasites to other white blood cells such as lymphocytes in the lymphoid tissues for example, thus participating to parasite dissemination and continuous immune stimulation (Kicia et al., 2018; Brdícková et al., 2020; Sak et al., 2021). These *in vivo* observations therefore reinforce the role of phagocytes, namely Mo, as Trojan horses for the dissemination of Microsporidia to other organs, in our newly set up model (Dalboni et al., 2021).

The mouse ear pinna model further enabled us to obtain reproducible quantitative data on motility parameters (speed, displacement, straightness) of recruited innate immune cells at the injection site. Using the Imaris software, stringent algorithm settings were applied, as previously described (Abdul Hamid et al., 2020, 2021). While analyzing high numbers of cells (Table 1), heterogeneity in results was observed and was the reflection of the complexity of innate immune responses that are set up at early time-points, depending on molecular signals (cytokines, chemokines), cell-parasite interactions or not at the injection site, contact time between cell and parasites, distance between cells and parasites at the injection site and phenotypes of recruited inflammatory cells. Analysis of innate immune cells migration showed that cells behaved differently in presence of parasites. Spores decreased cell speed at 2 h pi, allowing some recruited cells to interact with the inoculum. At this time point, the significant increase of the straightness parameter could be related to high numbers of “quick” cells not interacting or interacting very shortly with parasites to rapidly leave the injection site. At 3 days pi, modulation of cell motility parameters persisted, as speed was significantly increased, and straightness was decreased. These results suggest the potential diffusion of small molecules capable of influencing the behavior of recruited cells, in link with parasite maintenance in the skin.

Finally, we assessed the ability of *E. cuniculi* parasites to proliferate in the mice ear pinna model. According to literature, this microsporidian species completes a development cycle inside Mo and fibroblasts in 3 days (Han et al., 2019). A significant increase of the parasite load in the ear pinna would thus be expected at day 3 pi if the parasite could develop in this tissue. However, the parasite load was not measurable using the CW + signal intensity, as the newly formed chitinous wall is no longer labeled once the sporoplasm is released from the extruded spore (Table 1). Thus, the persistence of the CW + signal until day 7 pi could be related to the presence of empty spores after sporoplasm ejection, and didn’t necessarily mean that the parasite had completed new division cycles. We can’t also exclude that host innate immune responses contribute to spore devitalization and to obtain non-infectious « ghosts » of spores that remain at the injection site. To clarify this point, immunolabelings were performed on ear tissues cryosections of infected mice, from 2 h to 7 days pi. Increasing numbers of other parasite development stages were detected over time, with the presence of high numbers of infectious foci at days 5 and 7 pi. These observations were strongly in favor of the ability of *E. cuniculi* to develop and to proliferate in the ear pinna model. Complementary experiments are required to know if the potential newly produced spores are infectious, by visualizing extruded polar tubes in the ear tissue of infected mice for example. For the immune system, the maintenance of spore material and the presence of developing parasites at the injection site represents an important source of antigens that influences the initiation and the outcome of host immune responses.

The mice ear skin model proposed here measures in parallel the dynamics of the inflammatory responses induced by microsporidian parasites and their fate over time (Figure 7). It reproduces the development cycle of the parasite *in vivo* and offers many perspectives to increase our understanding of the innate and adaptive immune responses directed against Microsporidia, both in immunocompetent and immunocompromised hosts. It will also contribute to elucidate the mechanisms of immune hacking used by Microsporidia to persist in their host and to develop new preventive strategies to fight against Microsporidia infections in human.

## Data availability statement

The raw data supporting the conclusions of this article will be made available by the authors, without undue reservation.

## Ethics statement

This animal study was reviewed and approved by the Ethics Committee on Animal Experimentation Auvergne (C2E2A), Clermont-Ferrand, France Agreement number: 28868.

## Author contributions

EC, AA, IF, AD, FD, and PG conceived and designed the experiments. EC, AA, and IF analyzed the data. IF, EC, AA, and PG performed the experiments, participated in the provision of materials for experiments and verified the overall replication and reproducibility of results, visualized, and wrote the manuscript. PG supervised, managed, and coordinated responsibility for the research activity. All authors contributed to the article and approved the submitted version.

## Supplementary material

Supplementary Figure 1 Fluorescence observation of *E. cuniculi* inoculum after labeling with Calcofluor White (CW) (blue) and anti-*E. cuniculi* polyclonal serum (*Ec*) (red). A double immunolabeling Cw + *Ec* + corresponding to the sporal stage is observed in most cases. A small proportion of Cw-*Ec* + parasites (filled white arrows) correspond to the other stages of parasite development **(A)**. SEM micrograph of a CW-labeled spore **(B)** Scale bar: 2μm.

Supplementary Figure 2 Photographs of the ears of mice intradermally injected with PBS 24 h pi **(A)** or CW + *E. cuniculi* spores 24 h pi **(B)**, 48 h pi **(C)**, and 7 dpi **(D)**. No signs of inflammation are visible 24 h post-PBS injection. In infected mice, the injection sites are visible (black arrowheads). Vasodilation is observed, as well as punctiform hemorrhage areas that intensify at 48 h pi and persist until 7 days pi.

Supplementary Figure 3 Live confocal imaging after micro-injection of *E. cuniculi* PBS at 3 h50 **(A)** and spores **(B)** in the ear pinna of LysM-EGFP transgenic mice. Decomposition of a confocal microscopy video showing a recruitment of EGFP + phagocytic cells after the injection of PBS into the mouse ear pinna. Immune cells are present at the injection site, due to the trauma of intradermal injection with the 34G needle into the ear tissue [**(A)**, control]. Decomposition of a confocal microscopy video showing a massive recruitment of EGFP + phagocytic cells that arrive and stop at the inoculation area to interact with CW + parasites (empty white arrowheads). An internalized spore is detected inside an EGFP + phagocyte (filled white arrows). *n* = 8 tissues (*E. c*) and *n* = 14 control tissues (PBS). Scale bar: 50 μm.

Supplementary Figure 4 Confocal immunofluorescence images of immunolabeling cryosections of infected ear tissues. LysM-EGFP mice (EGFP + phagocytes: green fluorescence signal) were inoculated with fluorescent *E. cuniculi* spores (CW + : blue fluorescence signal). Immunostaining was performed on cryosections obtained from 48 h to 7 days pi **(A–D)**. Magenta, red, and blue fluorescence signals correspond to specific labeling of keratin (K5 mAb), all parasite stages (anti-*E. cuniculi* polyclonal serum), and cell nuclei (DAPI)/spores (CW), respectively. At 48 h pi, aggregates (insets) of mono-labeled parasites by *E.c* polyclonal serum are observed **(A)**. The size of these aggregates grows over time, highlighting developing infectious foci (insets) **(B–D)**. Intracellular infectious foci of *E. cuniculi* parasites inside fibroblast cells (HFF) at 72 h pi, after immunostaining with anti-*E. cuniculi* polyclonal serum. Infectious foci are in close proximity to cell nucleus (DAPI staining) **(E)**. Three independent experiments, *n* = 30 tissues (*Ec*) and *n* = 12 tissues (PBS). Scale bar: 10 μm or 2μm (insert).

Supplementary Video 1 EGFP + recruited phagocytes infiltrate the injection site after inoculation of PBS (control condition), due to the trauma caused by intradermal injection. *In vivo* confocal time-lapse imaging of phagocytes recruitment in LysM-EGFP transgenic mice ear tissue injected with PBS from 3 h 50 to 4 h 10 pi. Average projections of time-lapse images. Z-stacks collected 19 s apart. Scale bar: 50μm.

Supplementary Video 2 Numerous EGFP + phagocytes penetrate the injection site and interact with CW + *E. cuniculi* parasites of the inoculum. *In vivo* confocal time-lapse imaging of phagocytes recruitment in LysM-EGFP transgenic mice ear tissue injected with parasites from 4 h 15 to 4 h 35 pi. Average projections of time-lapse images. Z-stacks collected 7 s apart. Scale bar: 50μm.

Supplementary Video 3 Some recruited EGFP + phagocytes cross the injection site without or interacting quickly (“quick” phenotype) with CW + *E. cuniculi* parasites of the inoculum. *In vivo* confocal time-lapse imaging of phagocytes recruitment in LysM-EGFP transgenic mice ear tissue injected with parasites from 3 h 40 to 4 h pi. Average projections of time-lapse images. Z-stacks collected 24 s apart. Scale bar: 50μm.

Supplementary Video 4 Numerous EGFP + phagocytes penetrate the injection site and interact with CW + *E. cuniculi* parasites of the inoculum. Some parasites are detected intracellularly. *In vivo* confocal time-lapse imaging of phagocytes recruitment in LysM-EGFP transgenic mice ear tissue injected with parasites from 4 h 15 to 4 h 35 pi. Average projections of time-lapse images. Z-stacks collected 7 s apart. Scale bar: 50μm.
