# Supplementary figures and images for: A mouse ear skin model to study the dynamics of innate immune responses against the microsporidian Encephalitozoon cuniculi
Source: Front Microbiol. 2023 Apr 13;14:1168970. doi: 10.3389/fmicb.2023.1168970 (PMC10136781; doi:10.3389/fmicb.2023.1168970)

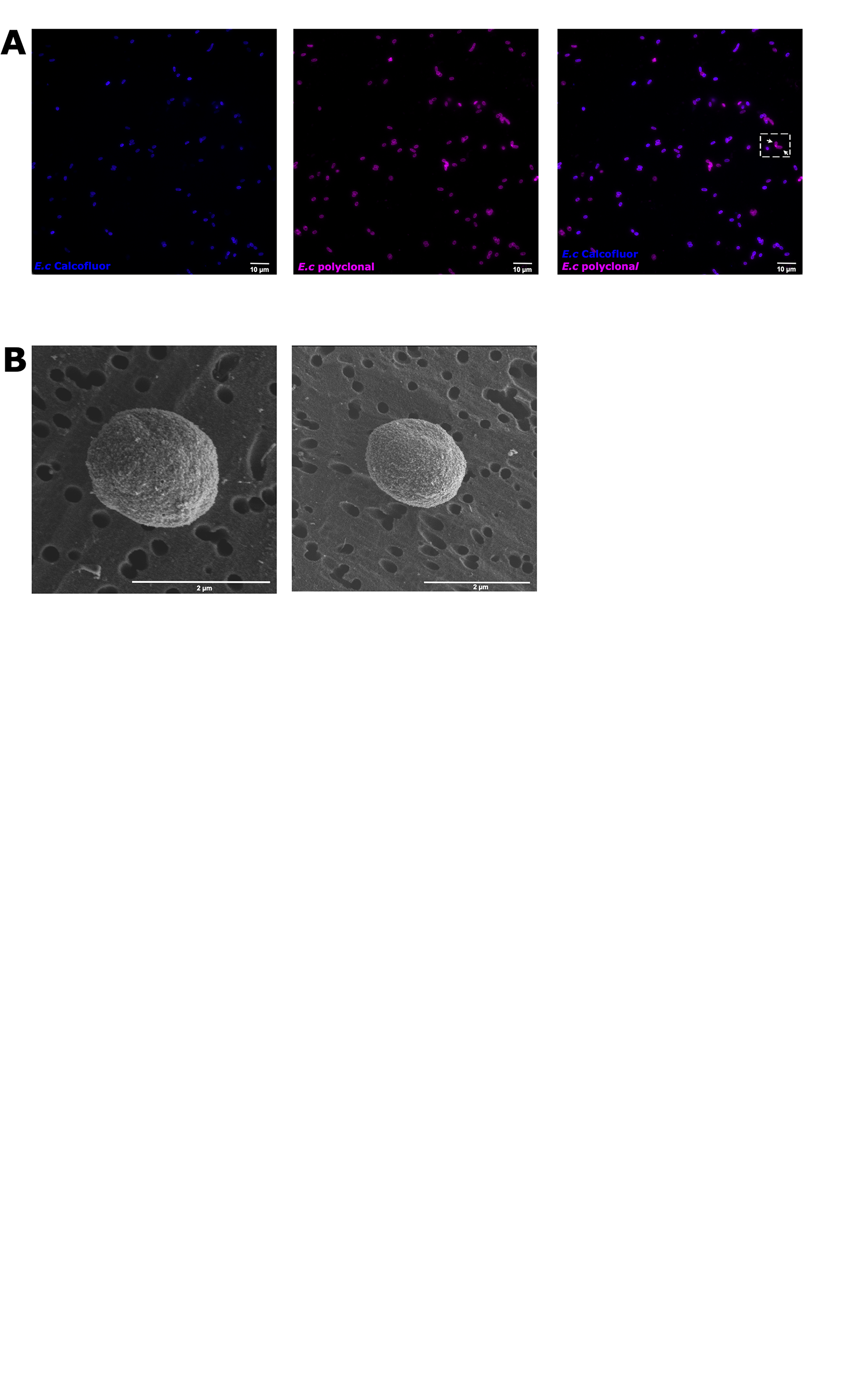

Supplement: Supplementary Figure 1 — Fluorescence observation of E. cuniculi inoculum after labeling with Calcofluor White (CW) (blue) and anti-E. cuniculi polyclonal serum (Ec) (red). A double immunolabeling Cw + Ec + corresponding to the sporal stage is observed in most cases. A small proportion of Cw-Ec + parasites (filled white arrows) correspond to the other stages of parasite development (A). SEM micrograph of a CW-labeled spore (B) Scale bar: 2μm. [file Image_1.TIFF]

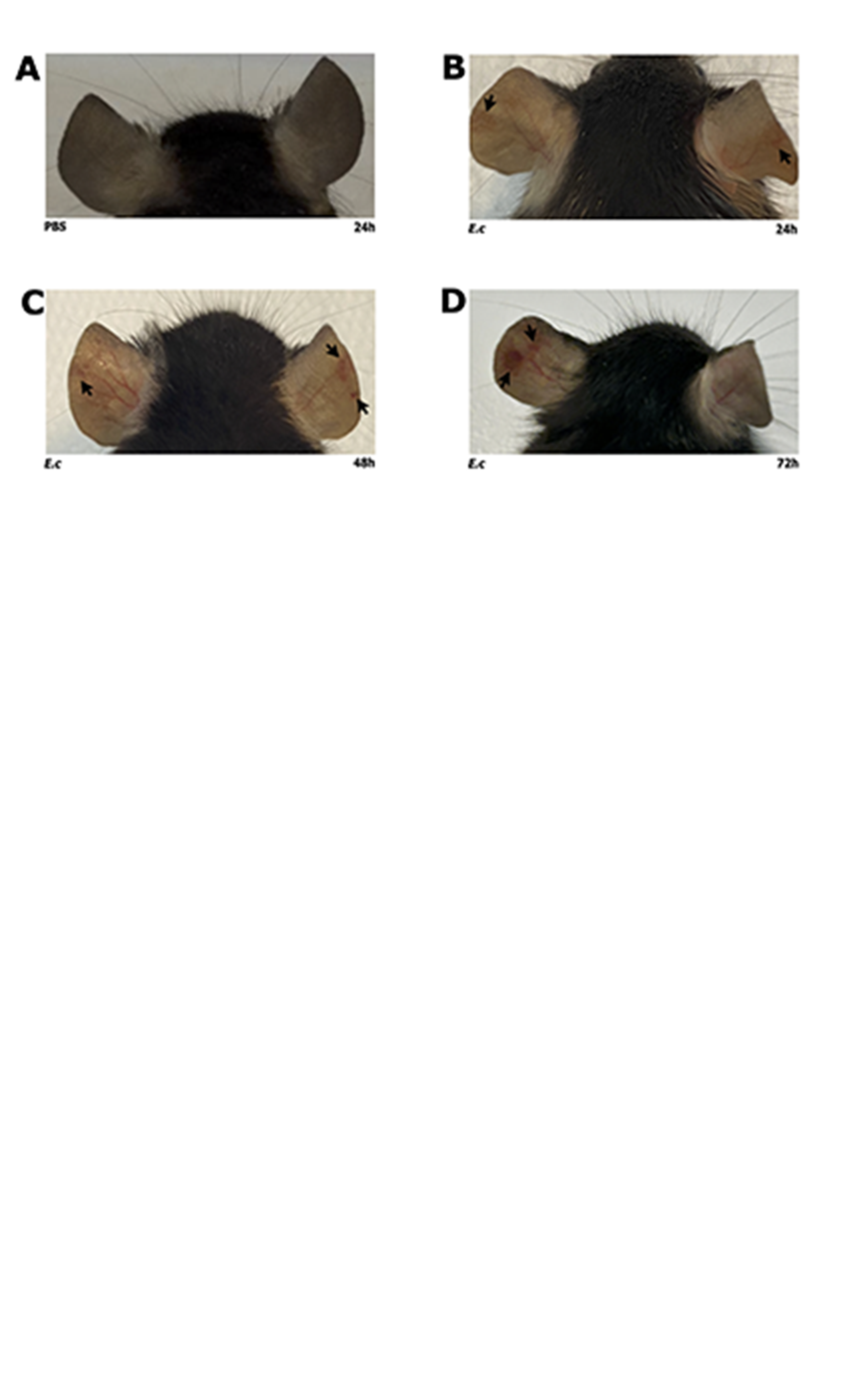

Supplement: Supplementary Figure 2 — Photographs of the ears of mice intradermally injected with PBS 24 h pi (A) or CW + E. cuniculi spores 24 h pi (B), 48 h pi (C), and 7 dpi (D). No signs of inflammation are visible 24 h post-PBS injection. In infected mice, the injection sites are visible (black arrowheads). Vasodilation is observed, as well as punctiform hemorrhage areas that intensify at 48 h pi and persist until 7 days pi. [file Image_2.TIFF]

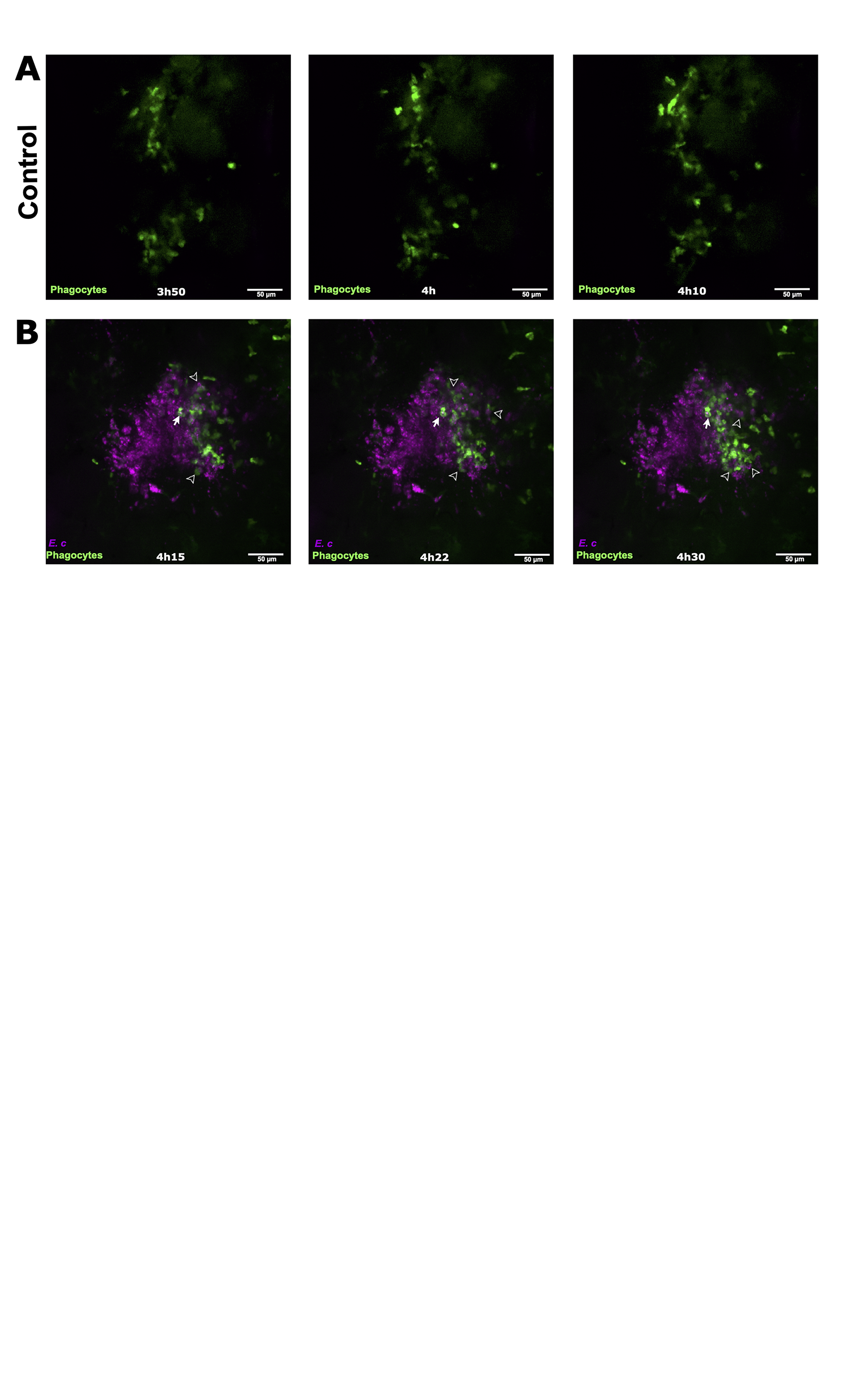

Supplement: Supplementary Figure 3 — Live confocal imaging after micro-injection of E. cuniculi PBS at 3 h50 (A) and spores (B) in the ear pinna of LysM-EGFP transgenic mice. Decomposition of a confocal microscopy video showing a recruitment of EGFP + phagocytic cells after the injection of PBS into the mouse ear pinna. Immune cells are present at the injection site, due to the trauma of intradermal injection with the 34G needle into the ear tissue [(A), control]. Decomposition of a confocal microscopy video showing a massive recruitment of EGFP + phagocytic cells that arrive and stop at the inoculation area to interact with CW + parasites (empty white arrowheads). An internalized spore is detected inside an EGFP + phagocyte (filled white arrows). n = 8 tissues (E. c) and n = 14 control tissues (PBS). Scale bar: 50 μm. [file Image_3.TIFF]

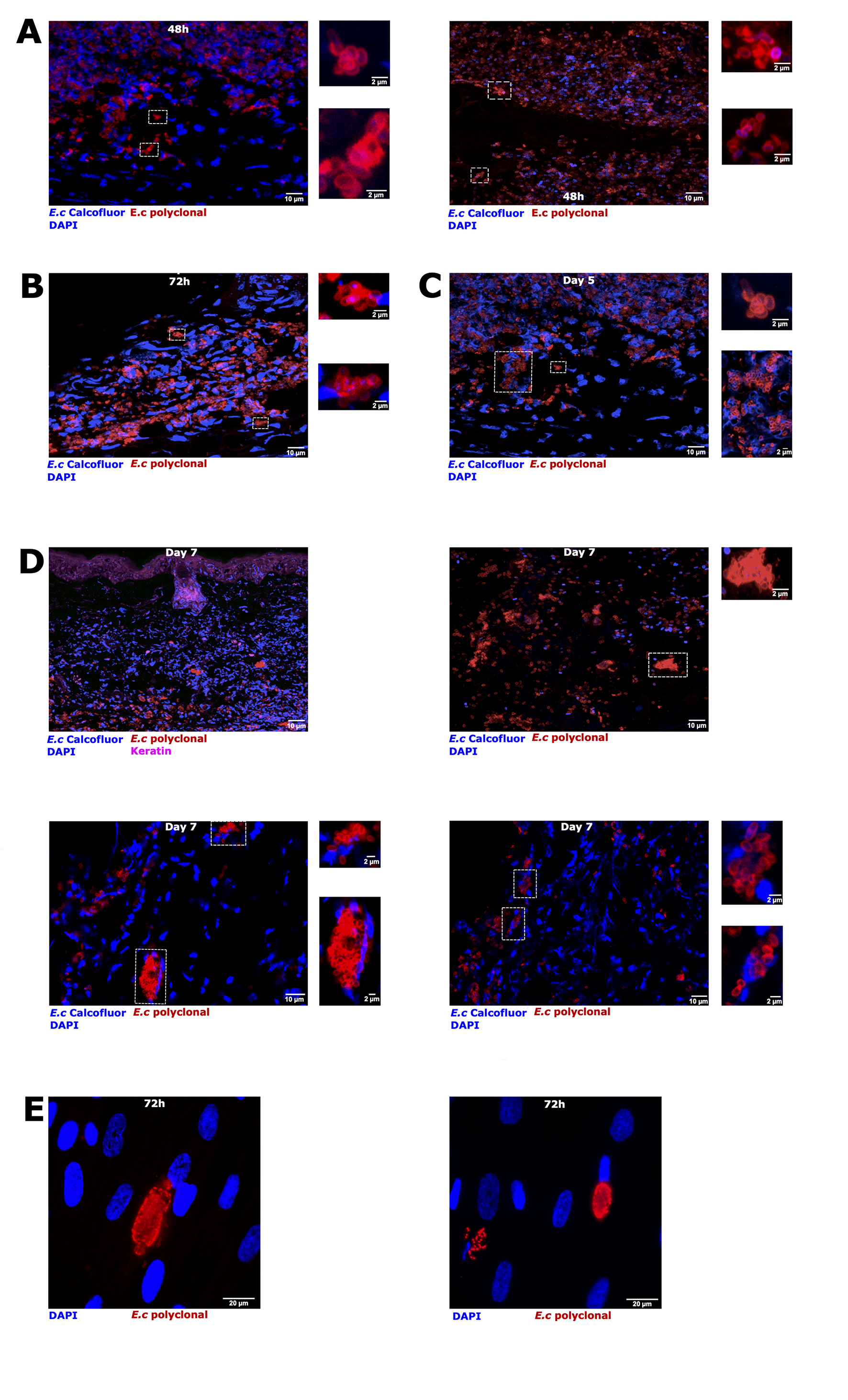

Supplement: Supplementary Figure 4 — Confocal immunofluorescence images of immunolabeling cryosections of infected ear tissues. LysM-EGFP mice (EGFP + phagocytes: green fluorescence signal) were inoculated with fluorescent E. cuniculi spores (CW + : blue fluorescence signal). Immunostaining was performed on cryosections obtained from 48 h to 7 days pi (A–D). Magenta, red, and blue fluorescence signals correspond to specific labeling of keratin (K5 mAb), all parasite stages (anti-E. cuniculi polyclonal serum), and cell nuclei (DAPI)/spores (CW), respectively. At 48 h pi, aggregates (insets) of mono-labeled parasites by E.c polyclonal serum are observed (A). The size of these aggregates grows over time, highlighting developing infectious foci (insets) (B–D). Intracellular infectious foci of E. cuniculi parasites inside fibroblast cells (HFF) at 72 h pi, after immunostaining with anti-E. cuniculi polyclonal serum. Infectious foci are in close proximity to cell nucleus (DAPI staining) (E). Three independent experiments, n = 30 tissues (Ec) and n = 12 tissues (PBS). Scale bar: 10 μm or 2μm (insert). [file Image_4.TIFF]
